# High-Sensitivity Seismometer Development for Lunar Applications

**DOI:** 10.3390/s23167245

**Published:** 2023-08-18

**Authors:** Leandro A. N. de Paula, Ronald S. Norton, Ho Jung Paik, Nicholas C. Schmerr, Paul R. Williamson, Talso C. P. Chui, Inseob Hahn

**Affiliations:** 1 Department of Physics, University of Maryland, College Park, MD 20742, USA; rsnorton@umd.edu (R.S.N.); hpaik@umd.edu (H.J.P.); 2 Department of Geology, University of Maryland, College Park, MD 20742, USA; nschmerr@umd.edu; 3 Austin Sensors, LLC, Austin, TX 78750, USA; roger@austinsensors.com; 4 Jet Propulsion Laboratory, California Institute of Technology, Pasadena, CA 91109, USA; talso.c.chui@jpl.nasa.gov (T.C.P.C.); inseob.hahn@jpl.nasa.gov (I.H.)

**Keywords:** lunar seismology, instrumentation, seismometer, planets, space

## Abstract

Lunar seismology is a critical area of research, providing insights into the Moon’s internal structure, composition, and thermal history, as well as informing the design of safe and resilient habitats for future human settlements. This paper presents the development of a state-of-the-art, three-axis broadband seismometer with a low-frequency range of 0.001–1 Hz and a target sensitivity over one order of magnitude greater than previous Apollo-era instruments. The paper details the design, assembly, methodology, and test results. We compare the acceleration noise of our prototype and commercial seismometers across all three axes. Increasing the test mass and reducing its natural frequency may further improve performance. These advancements in seismometer technology hold promise for enhancing our understanding of the Moon’s and other celestial bodies’ internal structures and for informing the design of future landed missions to ocean worlds.

## 1. Introduction

Lunar seismology has long been a subject of fascination and intrigue, not only for its scientific value but also for its implications for future human exploration and habitation of the Moon. Since the deployment of seismometers during the Apollo missions in the late 1960s and early 1970s, our understanding of the Moon’s seismic activity and interior structure has evolved significantly [1,2,3]. However, many questions and challenges still remain, warranting further investigation and the development of novel techniques to advance our knowledge of Earth’s natural satellite.

The primary goal of lunar seismology is to decipher the Moon’s internal structure, composition, and thermal history, which in turn can provide insights into the processes that have shaped its geological evolution [4]. Additionally, a comprehensive understanding of lunar seismic activity is crucial for designing and constructing safe, resilient lunar habitats for future human settlements [5,6]. In recent years, renewed interest in lunar exploration has brought forth innovative approaches to studying lunar seismology, including the development of advanced seismometers and the application of data analysis techniques adapted from terrestrial seismology [7].

These advancements have not only deepened our understanding of the Moon’s seismic activity and interior structure but also paved the way for the development of more sensitive and sophisticated instrumentation. One such innovation is the prototype two-horizontal-axis broadband seismometer for lunar applications, which our group developed under NASA’s Planetary Instrument Definition and Development Program (PIDDP) [8].

Building on this groundwork, as part of the Maturation of Instruments for Solar System Exploration (MatISSE) program, our team developed a three-axis broadband seismometer with a very low-frequency range of 0.001–1 Hz and a target sensitivity of at least 2 × 10^−10^ m s^−2^ Hz^−1/2^ at 1 mHz [9]. This seismometer aims at a sensitivity over one order of magnitude greater than those deployed during the Apollo era and is intended for deployment as part of a lunar geophysics network (LGN) or mission to an icy ocean world [10,11,12,13].

The over ten-fold enhancement in seismometer sensitivity across a bandwidth ranging from 1 mHz to 1 Hz enables the detection of remote teleseisms, examination of the Moon’s seismic background noise, and the potential identification of the Moon’s normal modes, such as the most pronounced spheroidal oscillation, 0S2, within the 8–12 mHz range [1,14]. These advancements will considerably improve the resolution of models of the Moon’s and other celestial bodies’ internal structures, thereby enriching our comprehension of their geological, thermal, and chemical composition [15,16,17]. 

The inner structures of ocean worlds, both shallow and deep, continue to be inadequately determined. A seismology experiment employing our advanced seismometer on a future landing mission could offer crucial insights into their planetary development. Through seismic monitoring of the subsurface, we can potentially identify fluid movement in the shallow layers, seismic signals originating from cryovolcanoes, and perhaps even sub-glacial ocean circulation [18].

In this article, we report the development of our MatISSE Planetary Broad-Band Seismometer (PBBS), carried out at the University of Maryland. We summarize the design and construction of our second prototype (PT-2), emphasizing the primary mechanical procedures involved in building the sensor head. We then discuss our procedure for test mass alignment and centering and present the test results of our three-axis PT-2 seismometer. Finally, we determine the Brownian motion noise level of the instrument. We show that, by replacing the test mass with one made with a higher-density material, PT-2 should be able to meet the LGN target sensitivity over the entire frequency range of 1 mHz to 1 Hz. 

## 2. Design and Construction

A comprehensive description of the hardware and its operating principles is given in Reference [19]. In this section, we summarize the transducer and circuit scheme. We also detail the sensor head construction, specifically the capacitor electrodes utilized for displacement capacitance sensing (DCS) and electrostatic frequency reduction (EFR). 

### 2.1. Seismometer Overview

**Figure 1:**
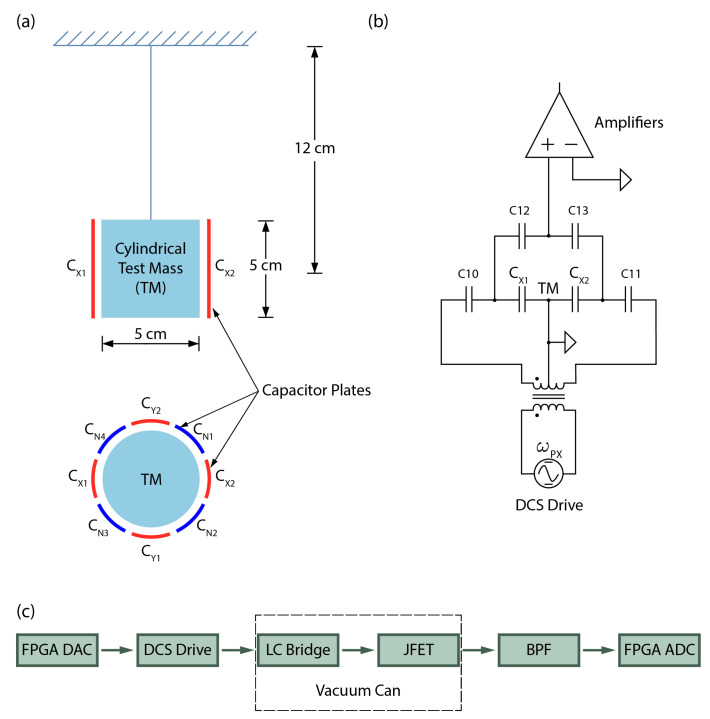
(**a**) Mechanical configuration of capacitor plates for the two horizontal axes employed for both sensing and frequency reduction in the seismometer. The test mass is suspended using springs and is surrounded by sensors and actuator electrodes. (**b**) Schematic circuit diagram for DCS for the x-axis. The LC resonant circuit formed by the two sensing capacitors and two equal inductors is driven by an oscillator at frequency ω_px_ ≫ω. The amplifier output is demodulated to obtain the low-frequency seismic signals. (**c**) Block diagram representing the signal path originating from the Digital Analog Converter (DAC) and processing through successive stages encompassing the DCS drive, the LC bridge, the JFET, and culminating in the band pass filter (BPF) before reaching the analog digital converter (ADC). The LC bridge employs AC-coupled drive and detection to minimize errors arising from the non-ideal transformer. Capacitors C10 and C11 provide AC drive coupling to the test mass and permit the DCS (Cx1 and Cx2) electrodes to float. Subsequently, the C12 and C13 capacitors work in tandem to provide the DCS signal with reference to ground. While this circuit design deviates from conventional approaches, the drive voltage can be commanded over a wide range, which allows compensating the loss from capacitive coupling of the output by increasing the low-impedance drive voltage. Notably, the ultimate sensitivity of the DCS is limited by the JFET amplifier input noise, rather than the drive amplitude.

The operational principle of a seismometer entails a mass-spring system characterized by an elastic or gravitational restoring force, as illustrated in Figure 1a. The three axes employ the same principles. Electrodes are strategically placed in the vicinity of the test mass to detect the displacement induced by ground motion. For sensing purposes, a DCS scheme is employed. Capacitors C_i1_ and C_i2_, where i (=x, y, z) labels the axes, form coupled pairs situated on opposite sides of the test mass. They are part of an LC bridge circuit that is driven by a frequency generator oscillator, as shown in Figure 1b. The seismic signal at frequency *ω* generates two sidebands at *ω*_px_ ± ω. The sensed signal is subsequently decoded using a digital lock-in amplifier.

In order to enhance the instrument sensitivity at low frequencies, capacitors C_Nj_ (where j = 1–6) are utilized for electrostatic frequency reduction (EFR). By applying a high voltage between these capacitor plates and the test mass, a negative spring effect is created, which effectively counteracts the effects of the positive elastic or gravitational spring, thereby significantly reducing the resonance frequency to approximately zero. Additionally, the EFR system was also designed with trim and offset functionalities, enabling fine-tuning adjustments.

**Figure 2:**
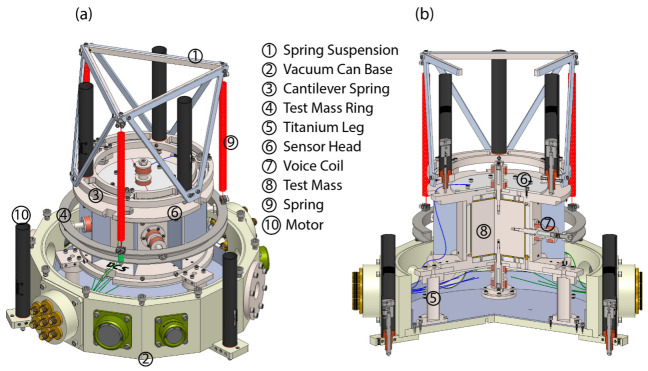
A perspective view of the hardware, encompassing the sensor head, springs, suspension, motors, and the lower section of the vacuum can. Notably, the representation intentionally excludes the cover of the vacuum for clarity and simplification. (**a**) The comprehensive depiction of the hardware assembly. (**b**) The 120-degree sectional view unveiling the internal components and structure of the instrument. Six screws are affixed to the test mass, each aligned along one of the three orthogonal axes. With magnets inside the screws and solenoids around the magnets, voice coils are formed and can be used for feedback.

Figure 2 presents an illustration of the internal hardware configuration within the vacuum can. The cylindrical titanium (Ti) test mass, enclosed within the electrode housing, is rigidly connected to an external Ti ring with four perpendicular horizontal Ti screws, which protrude through clearance holes in the electrodes, MACOR^®^ insulator, and Ti housing. The external ring is clamped to the lower ends of three helical tungsten (W) springs. The upper ends of these springs are clamped at the vertices of a Ti equilateral triangle, which is supported by a light, rigid triangulated aluminum (Al) frame structure from the load end of a Ti 2D folded beam spring plate, which can be raised, lowered, and tilted using three motorized linear actuators also attached to the load ring of the spring plate. The other ends of the spring plate beams are anchored to the top of the electrode housing at three places, and the actuators push against the top surface of the electrode housing. Outside the vacuum can, an additional three motorized linear actuators are utilized to adjust the horizontal position of the test mass by tilting the whole instrument.

### 2.2. Gap Spacing Construction

**Figure 3:**
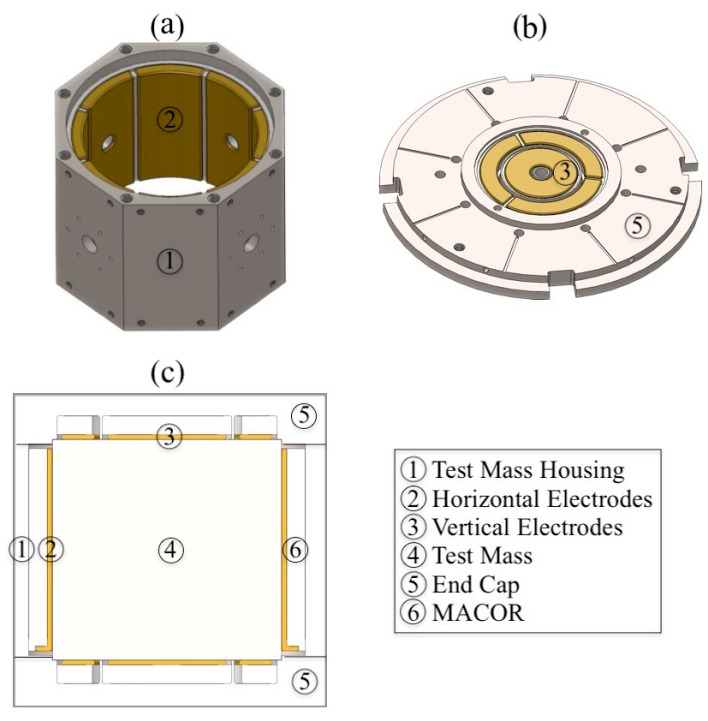
(**a**) and (**b**) are perspective views of the Ti housing and one of the end caps, and (**c**) is their cross-sectional view. The Ti test mass is also shown inside the Ti housing.

Within the housing, the test mass is almost completely surrounded by brass electrodes separated from the test mass surfaces by a nominal gap of 0.125 mm when the test mass is perfectly centered. The eight horizontal electrodes (Figure 3a) form an azimuthally segmented cylindrical shell with a 1 mm separation between the segments. These were originally machined in a single part with a 1 mm-thick non-segmented inner shell, which was later removed by wire EDM (Electrical Discharge Machining) after the whole part had first been epoxied into the insulating MACOR^®^ cylindrical shell, which was subsequently epoxied into the Ti housing. Teflon stoppers and spacers were used to precisely control part positioning and epoxy flow. Epotek^®^ 301-2 2.5 GM low-viscosity epoxy was used, with a 3 h curing time at 80 °C. Post-assembly cleaning, including spacer and stopper removal, was required.

The eight horizontal electrodes form four pairs of diametrically opposed electrodes. Two orthogonal pairs are used for DCS and define the horizontally sensitive axes. The other two pairs, also orthogonal but rotated 45 degrees from the sensitive axes, are used for EFR.

The procedure for constructing the top and bottom housing end caps was somewhat similar in that four separate brass electrodes, a single inner annular one and three forming a segmented outer annulus, were first epoxied to a MACOR^®^ disk, which was subsequently epoxied to the Ti end cap. The epoxied assembly was then precisely machined on a CNC milling machine to achieve the required flatness, parallelism, and vertical electrode separation from the test mass, as well as to ensure perpendicularity with the horizontal electrodes and housing.

Although bumpers were initially considered to prevent short circuits when the test mass contacted the electrodes, they proved unnecessary in practice. However, subsequent inspection revealed spot welding marks on the vertical electrodes, likely caused by high EFR electrical discharge in the gap spacing. No additional significant damage that might jeopardize the electrical integrity of the circuitry was observed. Electrical connections with the printed circuit boards (PCBs) on the end caps’ outer surface were facilitated by spring-loaded probes that contacted the electrodes through holes in the MACOR^®^ insulator and Ti end caps.

The springs suspending the test mass were manufactured out of “non-sag doped” W by Union City Filament Corp. Tungsten was chosen for its low thermal expansion coefficient and low internal friction [20].

## 3. Experimental Test

### 3.1. Setup

**Figure 4:**
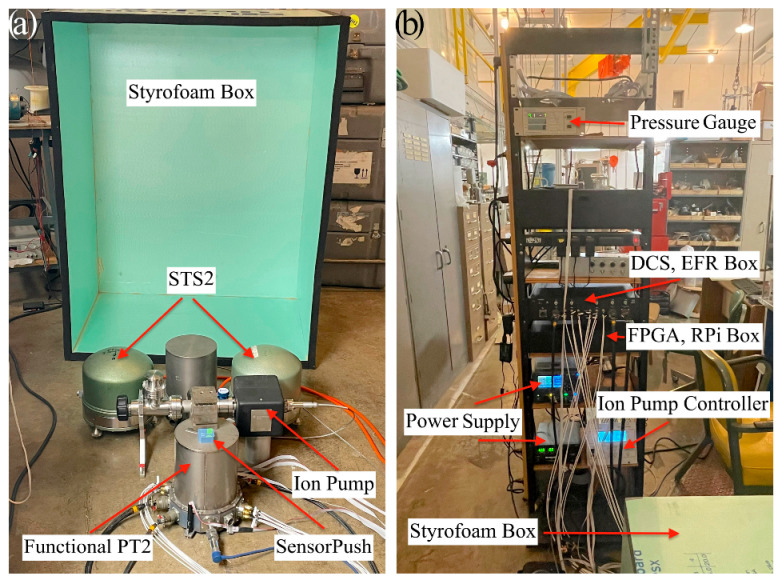
(**a**) Experimental setup showing the raised Styrofoam box, STS-2 and PT-2 seismometers, ion pump, SensorPush^®^ and vacuum plumbing. (**b**) Electronics rack showing the lowered Styrofoam box, power supplies, pressure gauge, EFR and FPGA boxes and ion pump controller.

Figure 4a shows the experimental setup for PT-2, featuring two PT-2 units, although only one is functional. The other unit consists solely of the vacuum can, without the seismometer hardware. Both units share the same vacuum plumbing, with an ion pump installed in a central vacuum valve. Indium served as the sealant for most joints, while rubber O-rings were retained for coaxial cable connectors, as the experiment was performed at room temperature. The resulting pressure achieved was approximately 1 µtorr.

To measure pressure and temperature, a sensor device (SensorPush^®^, Brooklyn, NY, USA) was positioned atop the operational seismometer. To minimize atmospheric disturbances, a thermally insulating Styrofoam box was constructed and placed over the hardware. The box effectively mitigated short-term atmospheric fluctuations but was unable to counteract more pronounced long-term variations. Nonetheless, it effectively suppressed sharp and fast changes.

All electronic components were supplied by our collaborator at Austin Sensors (see Figure 4b). The provided equipment included two electronic boxes, cables, a monitor, a computer, and a power supply. One box housed three field-programmable gate arrays (FPGAs) and three Raspberry Pis (RPIs), one per axis. The other contained PCBs for DCS, EFR, and general monitoring, assembled on a motherboard. The majority of components were available in triplicate, one for each axis. The boxes were electronically connected via cabling between the seismometer and the computer. Austin Sensors also developed user-friendly Python software and documentation, which are stored on a local disk.

Two STS-2 seismometers and a REFTEK RT130 data logger were borrowed from the Carnegie Institute and operated concurrently with the PT-2.

### 3.2. Procedure for Centering

A significant challenge in the experiment involved the freeing and centering of the test mass relative to the surrounding sensing electrodes. The gap spacing formed between the test mass and electrodes was a mere 0.125 mm, while the perpendicular surface dimensions facing the gap were 5 cm × 5 cm, yielding a 1:400 ratio of gap spacing to surface dimension. 

The process of freeing the test mass from contact with an electrode and achieving even spacing from all electrodes was accomplished through a two-phase procedure involving EFR in both off and on states. The procedure entailed a visual approach followed by an electronic approach with EFR off, and fine-tuning adjustments with EFR on.

During the visual approach, with the vacuum can open and the sensor head exposed, the spring height was adjusted by using screws at the spring clamp points. This allowed for the test mass to be suspended by the springs and, subsequently, its position to be assessed and adjusted to avoid contact with the electrodes. The inner and external motors help position the test mass, ensuring freedom of movement and approximate centering. The combination of mechanical and electronic adjustments facilitated the initial iteration of the process, with the test mass rotational mode observed to decay over a period of approximately five minutes. Modes affecting the gap spacing, such as vertical, horizontal, and rocking modes, were significantly damped and not observable due to atmospheric pressure in the gap. 

Upon sealing and evacuating the vacuum can, fine-tuning was conducted exclusively through electronic means. The test mass parallelism concerning the capacitor plates was achieved by executing a computer calibration script involving the six segmented vertical sensing electrodes. The calibration script was run each time the test mass was moved vertically by the internal motors to evaluate centering relative to each segmented electrode pair, positioned at the top and bottom. If the test mass is properly centered, an EFR voltage exerts equal and opposite forces on the test mass from the two opposing capacitor plates. Therefore, changing the EFR voltage will not cause the test mass to move. On the other hand, if the test mass is not centered, a change in the EFR voltage will cause the test mass to move to a different location. Hence, the objective of the centering mechanism is to move the test mass to a location where its displacement, as indicated by the DCS, does not change when the EFR voltage is altered. This position differed from the DCS center due to a mismatch in the stray capacitance. Using these processes, the resonance frequency was successfully reduced from 2.89 Hz to 0.5 Hz. 

With the vertical axis configuration maintained at 0.5 Hz or slightly higher, the EFR was next applied to the horizontal axes. Due to the complexities of working with two axes in the horizontal plane, the test mass could not be positioned at the EFR center. Consequently, the horizontal axes were centered at the DCS center by using the external motors. Gradual and non-isotropic EFR applications enabled the reduction in the two horizontal modes from 1.5 Hz to 0.5 Hz. High-quality factors observed in all three directions indicated that the test mass was successfully freed. 

## 4. Data Analysis

### 4.1. PT-2 Transfer Function

The STS-2 seismometer exhibits a flat response between 120 s and 50 Hz, with ground motion velocity data derived through a multiplicative factor applied to the raw data [21,22]. Acceleration noise is subsequently calculated using a derivative calculus procedure on the velocity dataset. As the PT-2 response to ground motion has not been experimentally determined, a theoretical derivation is employed. The following equation presents the transfer function for a mass-spring system [23]:

(1) $$\left|H\left(\omega \right)\right|=\sqrt{{\left(\omega_{0}^{2}-\omega^{2}\right)}^{2}+{\left(\frac{b}{m}\omega +\omega_{s}^{2}\phi_{i}\right)}^{2}},$$

where *b* represents a parameter determining the system’s viscous damping, considered null (*b* = 0) in our case, *m* is the mass of the test mass; *ω* is the ground motion frequency; *ω_s_* and *ω*_0_ denote the system’s natural and reduced frequencies, respectively. On the other hand, the loss angle due to the spring’s internal friction can be expressed as a function of the quality factor *Q*_i_ associated with that channel [24]:

(2) $$\phi_{i}=\frac{\omega_{0}^{2}}{\omega_{s}^{2}}\frac{1}{Q_{i}}.$$

Substituting Equation (2) into Equation (1) yields the transfer function in terms of the quality factor computed when PT-2 is operated at the reduced frequency *ω*_0_:

(3) $$\left|H\left(\omega \right)\right|=\sqrt{{\left(\omega_{0}^{2}-\omega^{2}\right)}^{2}+\frac{\omega_{0}^{4}}{Q_{i}^{2}}}.$$

The ground motion acceleration noise, presented in the next section, is determined by multiplying the transfer function by the PSD of the test mass displacement raw data obtained from the experiment. 

### 4.2. Power Spectral Densities 

**Figure 5:**
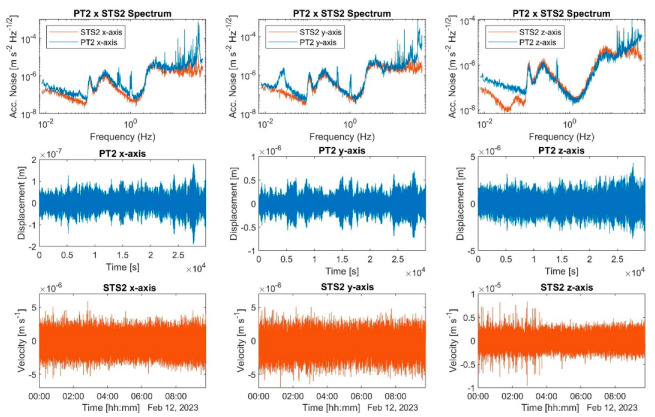
Plots from data with EFR applied on all three axes simultaneously. The graphical representations are organized in a three-column format, with each column corresponding to one of the axes. In the first row, a comparative analysis of the acceleration noise spectral densities for PT-2 and STS-2 is illustrated. The second row shows the temporal evolution of test mass displacement, while the ground motion velocity time series is seen in the third row. The peak at 0.5 Hz represents the resonance frequency of the test-mass-spring system, while the frequencies at 27 mHz and 1 Hz account for crosstalk.

Figure 5 presents data plots derived from all three axes, with EFR applied simultaneously. Each axis had a sample rate of 250 samples per second, which was the same as that for the STS-2. The DCS drive amplitude was set to 0.15 V. The first column displays the x-axis spectrum and time series for both PT-2 and STS-2. A cluster of peaks ranging from 0.46 Hz to 0.54 Hz is observed, potentially attributable to frequency drift and closely positioned horizontal modes. We employed the average frequency in the transfer function, suppressing the 0.5 Hz frequency peak with a Q-factor of approximately 150. Additional peaks near 1 Hz and 0.1 Hz are visible, possibly resulting from axes crosstalk and ground motion, respectively. The y-axis spectrum bears similarities to the x-axis spectrum, save for an isolated peak at approximately 27 mHz, which might also stem from crosstalk. 

The z-axis component spectrum exhibits poor agreement with the STS-2 spectrum at frequencies below 86 mHz, where the PT-2 acceleration noise was 5.8 × 10^–8^ $m\;s^{–2}Hz^{-1/2}$. The STS-2 minimum noise occurred at 39 mHz with a value of 1.05 × 10^−8^ $m\;s^{-2}Hz^{-1/2}$. This discrepancy amounts to a factor of roughly 5.5 between PT-2 and STS-2 at low frequencies. Due to data filtering, the time series of all three axes did not display significant displacement drift during the examined data set period, which is a segment of a 24 h-long data collection run characterized by reasonable test mass stability before eventual collapse. As predicted by our EFR theory, the test-mass energy potential well is relatively shallow at this frequency level, rendering the test mass prone to losing stability by escaping the potential well due to environmental noise-driven kinetic energy.

**Figure 6:**
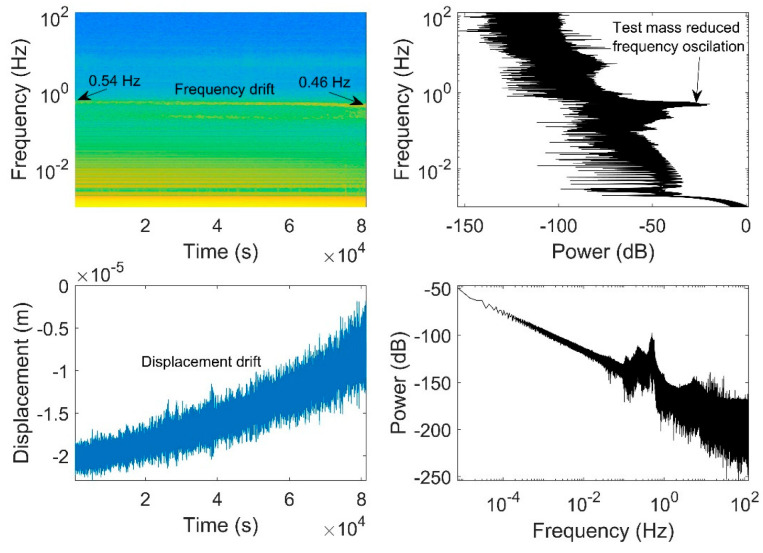
The spectrogram of vertical axis is shown in the top left quadrant with three more auxiliary plots. Bottom left: the test mass displacement is presented as a time series. Top right: the reciprocal of the power of displacement as a function of frequency, which is shown in the bottom right. The magnitude of the spectral lines in the top-left region of the graph can be observed by tracing horizontally towards the right, where the highest magnitudes are depicted as peak values.

Figure 6 presents the spectrogram of test mass displacement for the vertical axis alongside the unfiltered displacement data as a function of time, corresponding to the filtered data depicted in Figure 5. Additionally, the power of displacement and frequency are plotted against each other. The unfiltered displacement graph clearly reveals drift over time. In the spectrogram, it is evident that the entire spectrum, from the pendulum frequency of about 0.5 Hz downward, originates from a continuous signal. No distinct signals, such as a chirp—which may result from an earthquake—are observable within this time window.

The bottom left quadrant highlights the observable drift in displacement, while the top left quadrant concurrently illustrates the corresponding drift in frequency at around 0.5 Hz. The observed drift is likely a result of the combined effects of temperature drift and spring creep. However, no specific measurements have been conducted to ascertain the magnitudes of both factors at present.

### 4.3. Dynamic Range

**Figure 7:**
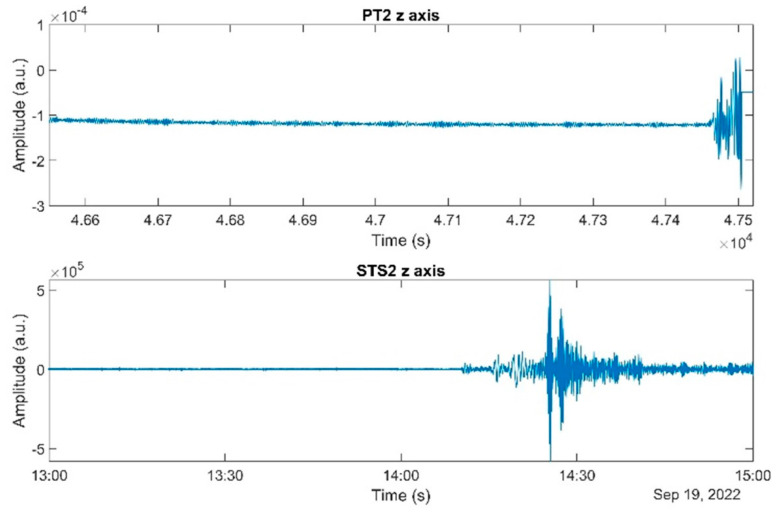
Earthquake detection data from the PT-2 and STS-2 instruments, collected on September 19, 2022. The upper panel presents the response of the PT-2 z-axis test mass displacement, while the lower panel depicts the ground motion velocity recorded by the STS-2 z-axis. Notably, the PT-2 instrument’s data for the entire earthquake event is absent, attributable to its limited dynamic range at the time. The PT-2 data have been truncated on the right side since they do not align with the scenario when the test mass is in a free state.

On 19 September 2022, pronounced oscillations were observed in real time on the PT-2 monitor screen. Upon investigating the signal on STS-2, it was confirmed that the signal represented genuine ground motion. See Figure 7. This event corresponded to a magnitude 7.6 earthquake off the Western Coast of Mexico, according to data available on the referenced website [25]. Due to the lack of a dynamic range implementation at the time, PT-2 could not record the entire signal duration as STS-2 did. The test mass lost stability while EFR was active and became latched to the electrodes. 

This incident prompts a discussion on the potential incorporation of dynamic range mechanisms. EFR applications reduce the frequency to enhance sensitivity at lower frequencies, allowing the detection of moonquakes with minimal amplitude at these frequencies. However, if the moonquake amplitude is sufficiently high, enhanced sensitivity is no longer required for detection. Consequently, it is feasible to deactivate EFR when the dynamic range is about to be exceeded, effectively providing a path for implementing a high dynamic range.

## 5. Brownian Motion Noise for the Vertical Axis

Part of the Brownian motion noise arises from spring internal friction. Although the current performance of the PT-2 vertical sensor is not solely limited by the Brownian motion noise of the suspension spring, it provides the ultimate and most stringent limit among all noise sources. We now explore potential strategies to minimize the Brownian motion noise. A formula presented in [23] defines the Brownian motion acceleration noise for a mass-spring oscillator as

(4) $$A\left(\omega \right)=\frac{1}{m}\sqrt{\frac{4k_{B}Tk_{s}\phi }{\omega }}={\left(\frac{4k_{B}Tk_{s}\phi }{{2\pi m}^{2}}\right)}^{1/2}\frac{1}{f^{1/2}},$$

where the parameter values for PT-2 are *m* = 0.465 kg, $k_{s}\equiv m\omega_{s}^{2}=$152 N/m, *T* = 293 K, and *k_B_* is the Boltzmann constant. The loss angle (*ϕ*) is material dependent and can be found by using the EFR technique [24]. A formula involving the quality factor and the reduced frequency is given by Equation (2):

(5) $$Q=\frac{1}{\phi }{\left(\frac{\omega_{0}}{\omega_{s}}\right)}^{2}=\left(\frac{1}{\phi f_{s}^{2}}\right)f_{0}^{2},$$

**Figure 8:**
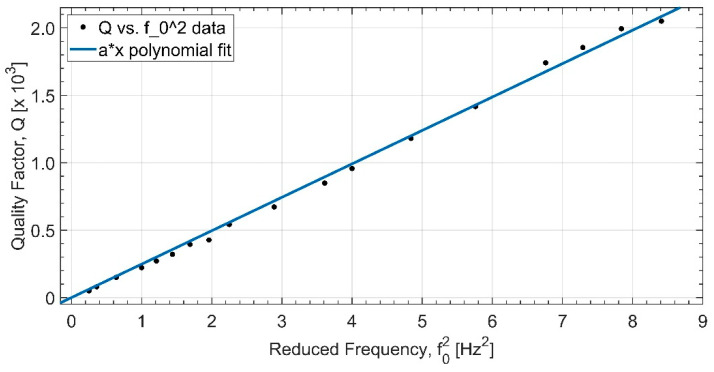
Experimental data for the *Q* factor are plotted against reduced frequency squared for the vertical axis.

Our experimental data for *Q* values are plotted against $f_{0}^{2}$ in Figure 8. A linear fit to the data yields

(6) $$Q=\left(247.9[s^{2}]\pm 1.7\%\right)f_{0}^{2}.$$

From this, we obtain the loss angle:

(7) $$\phi =4.8\times 10^{-4}.$$

Substituting Equation (7) into Equation (4), we calculate the Brownian motion noise for the vertical axis at *f* = 1 mHz for two different sets of *T* and *m*:

1. At earth room temperature, *T* = 293 K, *m* = 0.465 kg (Ti): 9.32 × 10^–10^ m s^−2^ Hz^−1/2^;
2. At lunar night-time temperature *T* = 140 K, *m* = 1.5 kg (Cu25-W75): 2.0 × 10^–10^ m s^−2^ Hz^−1/2^.

**Figure 9:**
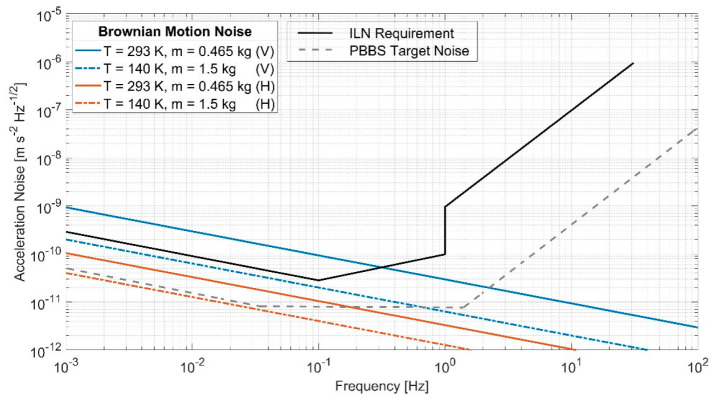
Comparative analysis of noise levels between the Brownian motion and the specifications set by the International Lunar Network (ILN). The figure shows two distinct configurations for both vertical and horizontal axes, based on varying temperature and mass parameters. The use of a test mass of 1.5 kg on the lunar surface appears promising for mitigating the Brownian noise, thus potentially allowing the system to achieve the sensitivity levels required by the ILN. V and H are abbreviations for vertical and horizontal axes, respectively.

We can see that, if the mass is increased from the present value, 0.465 kg, to *m* = 1.5 kg (by changing the test mass material from Ti to copper–tungsten, Cu–W), PT-2 could meet the stringent ILN requirement of 2.0 × 10^–10^ m s^–2^ Hz^–1/2^ at 1 mHz, as shown in Figure 9. Furthermore, the Brownian motion noise associated with the horizontal axes is lower than that for the vertical axis. This is because, for the horizontal axes, the stiffness is provided by gravity potential and EFR, which are both frictionless. There is only a small coupling to the internal damping of the spring material through the bending of the suspension spring near the suspension point [26]. Substituting the design parameters of our W spring into Equation (21) of [26], we obtain a dilution factor of 0.05 for damping, which leads to the estimated effective loss angle for PT-2 horizontal of $\phi_{e}=0.05\;\phi$, which was used to estimate the horizontal Brownian motion noise level. 

Note that the calculation of the loss angle ϕ required an extensive data collection of Q values while we employed the EFR technique to progressively reduce the vertical resonance frequency. Using this actual loss angle value, the effective loss angle $\phi_{e}$ is estimated and used to account for the losses associated with the horizontal degrees of freedom. The EFR technique applied to the horizontal motion is not employed to compute the horizontal Brownian motion.

## 6. Discussions

The spectrum for the PT-2 horizontal components exhibited two additional resonant peaks at frequencies of 27 mHz and 1 Hz. Since these peaks did not correspond to any features in the STS-2 spectrum, it is likely that they originated from instrumental sources. One plausible explanation is a potential disparity between the x- and y-axis FPGA clocks, which could generate slightly different drive frequencies and lead to crosstalk. However, the clocks have been replaced, and a more viable explanation is that these peaks were a result of the subtraction and addition of the two horizontal modes due to nonlinearities. We attempted to anisotropically adjust the horizontal EFR voltages in order to bring down the two horizontal modes from 1.5 Hz to approximately 0.5 Hz. Nevertheless, when these modes approached proximity, they could couple and give rise to split modes separated by 20 mHz around 0.5 Hz. This phenomenon could account for the presence of the additional two peaks through the subtractive and additive interactions of the two original modes.

Seismometers can experience deviations from linear behavior. The phenomenon of anharmonic oscillations reveals a complex interplay between nonlinearity and sensitivity. If the instrument operates within the nonlinear regime, anharmonic resonance could be triggered by a driving force whose frequencies do not match the natural frequency. Such oscillations can contribute to noise in the seismometer’s measurements, reducing the accuracy and reliability of the data obtained from the instrument. 

We were able to collect the data with resonance frequencies reduced to 0.2 Hz for the horizontal axes and 0.3 Hz for the vertical axes. However, as the frequency decreases, the test mass becomes more susceptible to instability. The potential well is shallower, and there is an increased likelihood of the test mass latching onto the electrodes. This problem will largely be alleviated in the quiet lunar environment. The data collected with lower resonance frequencies are not yet relevant, so the presented data corresponds to the more stable case where the resonance frequencies were approximately 0.5 Hz. A better agreement with STS-2 is anticipated upon controlling the test mass at lower frequencies, down to 0.01 Hz. 

The spectrum of all three axes exhibited a similar 1/f noise limitation, reaching nearly the same noise floor. This limitation in the PT-2 spectrum can be attributed to several factors. First, our inability to reliably decrease the resonance frequency from 0.5 Hz to the desired 0.01 Hz contributes to this limitation. Secondly, there may be other sources of noise, such as electronic noise, impacting the noise. In the case of an imbalanced bridge circuit, for instance, oscillator noise can arise, hindering the decoding of low-frequency sidebands. Third, the signal-to-noise ratio (SNR) is contingent upon the amplitude of the DCS drive, and the difficulty of maintaining the test mass near its central position at present prevents the utilization of the maximum drive amplitude level. As a consequence, the SNR is compromised, which is likely a contributing factor to the subpar performance observed at lower frequencies.

The DCS drive utilized in the system operates at a frequency of 40 kHz and is precisely controlled by a crystal. It exhibits minimal long-term drift at the scale of seconds. The variability in phase jitter primarily stems from the SNR. Prior to delivery, the bridge was fully balanced. However, it is uncertain whether any verification was conducted subsequent to installation. It is also plausible that the system may have been operational without the phase being appropriately configured. An additional factor that can introduce nonlinearity is the overdrive of the DCS resulting from setting the amplitude too high. The resulting drift of equilibrium position and off-center operation could have contributed to our experimental outcome. 

The pressure fluctuations may have an impact on the noise. This phenomenon is widely recognized within the field of seismology. However, we regrettably did not examine the correlation between PT-2 data and the corresponding pressure measurements. 

A temperature-dependent spring constant that changes over time can potentially lead to parametric resonance. When the spring constant of a system is modulated due to temperature changes, it can cause the system’s natural frequencies to vary over time as well. If the rate of temperature change and the modulation frequency align with certain modes of vibration, parametric resonance can occur. The temperature variations act as a periodic force that synchronizes with the natural frequency, causing the amplitude of vibration to increase significantly. Despite the installation of multiple thermometers positioned within the vacuum chamber, the internal temperature within the instrument was not monitored during the course of this measurement iteration. 

We have previously provided an extensive analysis of temperature-sensitivity-induced noise in seismometers, employing both quantitative and analytical approaches to explore the temperature dependence of a spring-suspended mass model with EFR [19]. We proposed practical solutions to minimize thermal effects on lunar seismometers by carefully selecting materials for the seismometer housing and spring suspension to balance shear modulus and thermal expansion coefficients. Our current configuration requires a distance between the top and bottom spring clamps of 105–108 mm to achieve a minimum of 99% cancellation/mitigation (Equation (39) in [19]). This would result in a temperature sensitivity of 2 × 10^−8^ m K^−1^ and a temperature spectral density of 30 µK Hz^−1/2^, meeting the ILN requirement of 2 × 10^−10^ m s^−2^ Hz^−1/2^ at 1 mHz. Employing a multi-layer insulation (MLI) shield may provide adequate temperature control on the lunar surface.

## 7. Conclusions

We tested our MatISSE PT-2 seismometer alongside two STS-2 seismometers and conducted a comparative analysis of acceleration noise between both instruments across all three axes. First, we applied EFR to the horizontal components while maintaining the vertical component in a suppressed state to avoid crosstalk. Second, we applied EFR solely to the vertical component. Finally, we ran all three axes concurrently, applying EFR and collecting data from all axes simultaneously. The comparison reveals that EFR can be implemented across all three axes simultaneously, and the PT-2 seismometer can operate without increased noise, barring potential electronic crosstalk or nonlinearities.

All three axes reached about the same noise floor at low frequencies. Although the main cause for this remains unclear, it is suggestive that all three axes are limited by the same 1/f noise at present. The PT-2 horizontal axes displayed better noise agreement with the STS-2 than the vertical axis. However, it is important to highlight that the vertical axis of the STS-2 seismometer exhibits greater sensitivity compared to its horizontal axes. 

EFR was applied to all three axes up to a reduced frequency of approximately 0.5 Hz. The observed Qs of 2000 and 12,000 for the vertical and horizontal components indicate that the test mass was free. Although the PT-2 has yet to surpass the STS-2’s low-frequency performance, operating all three axes at a reduced frequency of 0.5 Hz is considered an accomplishment, given the small gap spacing of 0.125 mm between the test mass and electrodes. To align with the STS-2 z-axis sensitivity, a possible solution entails lowering the EFR frequency to 0.01 Hz, which necessitates precise compensation of temperature sensitivity and exceptional test mass centering to avoid instability under high EFR voltage. 

Temperature-sensitivity-induced noise relies on effective temperature compensation and control. A noise level as low as 2 × 10^−10^ m s^−2^ Hz^−1/2^ at 1 mHz can be attained through spring height adjustments and the implementation of an MLI shield. Here, the Brownian noise was calculated using the experimental Q data. Increasing the test mass to approximately 1.5 kg by replacing Ti with Cu–W, while preserving PT-2’s other design parameters, may suffice to reduce the Brownian motion noise to ILN-required levels.

## Data Availability

The data that support the findings of this study are available upon reasonable request.
